# Bioinformatic Analysis of the Effect of Silver Nanoparticles on Colorectal Cancer Cell Line

**DOI:** 10.1155/2022/6828837

**Published:** 2022-04-11

**Authors:** Fernando Martínez-Esquivias, Melva Gutiérrez-Angulo, Julieta Saraí Becerra-Ruiz, Luz Andrea Martinez-Perez, Claudia Jackelin de la Cruz-Ahumada, Juan Manuel Guzmán-Flores

**Affiliations:** ^1^Instituto de Investigación en Biociencias, Centro Universitario de Los Altos, Universidad de Guadalajara, Tepatitlán de Morelos, Jalisco, Mexico; ^2^Departamento de Ciencias de la Salud, Centro Universitario de Los Altos, Universidad de Guadalajara, Tepatitlán de Morelos, Jalisco, Mexico

## Abstract

Colorectal cancer (CRC) is the most diagnosed cancer with the highest mortality rate each year globally. Although there are treatments for CRC, the development of resistance to therapies decreases the success of treatments. *In vitro* studies using the Caco-2 cell line have revealed the anticancer properties of silver nanoparticles (AgNPs) as a possible treatment for this disease. This study considered four researches that evaluated the proteomic profiles of cells of the Caco-2 line exposed to AgNPs. We performed a bioinformatics analysis to predict protein-protein interaction, hub genes, Gene Ontology (molecular function, biological process, and cellular components), KEGG pathways, analysis of expression, and immune cell infiltration. For these analyses, the STRING, DAVID, UALCAN, GEPIA2, and TISIDB databases were used. The results in Gene Ontology show that AgNPs cause a deregulation of genes related to cell-cell adhesion, the cytoplasm, the centriole, and carbon metabolism. Hub genes were identified, including *GADPH*, *ENO1*, *EEF2*, and *ATP5A1*, which showed differential expression in patients with adenocarcinoma of the colon and rectum. Additionally, the expression of the hub genes and immune cells was correlated. It was found that *ATP5A1* and *ENO1* were positively correlated with the infiltration of CD4+ T lymphocytes in colon adenocarcinoma and a negative correlation between *GADPH* and *PDIA3* with the infiltration of NK cells and CD4+ T lymphocytes in rectal adenocarcinoma, respectively. In conclusion, the administration of AgNPs causes an alteration of biological processes, cellular components, metabolic pathways, deregulation of hub genes, and the activity of immune cells leading to a potential anticancer effect.

## 1. Introduction

According to the American Cancer Society (ACS), colorectal cancer (CRC) is a type of cancer that originates in the colon or rectum. It is characterized by an abnormal growth of cells that clump together in the inner lining of the colon or rectum [1]. The abnormality in cell growth is due to genetic and epigenetic alterations that activate oncogenes and the inactivation of tumor suppressor genes [2]. Inflammatory bowel disease, family history, advanced age, obesity, smoking, alcohol intake, and consumption of red meat are some risk factors associated with the development of this disease [2–4].

CRC has become a health problem around the world. In 2020, the Global Cancer Observatory (GLOBOCAN) database reported that CRC ranks third in new cases diagnosed with 1,931,590 cases and ranks second in the highest number of deaths, registering 935,173 deaths from this disease [5]. Currently, the treatments available for CRC are surgery, chemotherapy, radiation, and targeted therapy. Implementing any of these treatments will depend on the stage, location, and degree of cancer that the patient presents [6]. However, these treatments are usually ineffective due to a late diagnosis and the development of resistance mechanisms to chemotherapeutic drugs and radiotherapy. Due to these limitations, there has been a continuous effort by the scientific community to develop new anticancer agents [7–9]. Silver nanoparticles (AgNPs) could be used for the treatment of cancer and in formulations to deliver anticancer drugs [10].

Moreover, recently, in vitro studies have demonstrated the anticancer potential of AgNPs in colorectal cancer cell lines. A study by Zein et al. [11] evaluated the anticancer effect of AgNPs synthesized using the extract of *Eucalyptus camaldulensis* leaves that were administered in the Caco-2 cell line. The results showed the highly cytotoxic effect on the cell line at low concentrations. However, a study by Khan et al. [12] synthesized AgNPs using the *Heliotropium bacciferum* plant extract and which were administered in the HCT-116 cell line and revealed that the administration of AgNPs led to cell apoptosis, an antiproliferative effect, and antimetastasis. Likewise, Dehghanizade et al. [13] reported that AgNPs synthesized using *Anthemis atropatana* extract had a dose-dependent cytotoxic effect and that administration led to apoptosis of the HT-29 cell line. Therefore, current research opens the door on the use of AgNPs as a potential therapeutic agent against colorectal cancer. However, integrative analyses are needed to help elucidate the anticancer mechanisms of AgNPs.

This study focuses on understanding the effect of AgNPs on the Caco-2 cell lines through *in silico* analysis. Four experimental studies that met defined search criteria were selected and analyzed, and different studies were implemented using bioinformatics databases to elucidate their effect.

## 2. Methodology

### 2.1. Literature Search

**Figure 1:**
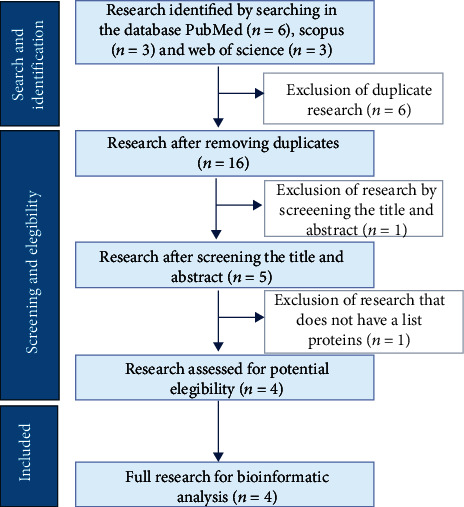
Guidelines and criteria for the selection of literature.

We searched in three databases: PubMed, Scopus, and Web of Science. The search was performed using the following terms: “Silver,” “Nanoparticles,” “Caco-2,” “Proteomic,” “Proteome,” and “Protein.” Additionally, the guidelines and criteria shown in Figure 1 were followed.

The titles and summaries of each of the articles were independently examined. The authors subsequently read and evaluated the complete text, and the relevant results of the proteomic analysis for each investigation were retrieved for further review and analysis.

### 2.2. Inclusion and Exclusion Criteria

The inclusion criteria for this study are (1) *in vitro* experimental studies; (2) Investigations that analyze the Caco-2 cell line proteomic profiles exposed to silver nanoparticles (AgNPs); (3) methods of assays used included nano-UHPLC-Orbitrap MS/MS unlabeled assay, tandem mass spectrometry (LC-MS/MS), protein identification by MALDI TOF-TOF MS/MS, two-dimensional electrophoresis, and gel electrophoresis of polyacrylamide with sodium dodecyl sulfate (SDS-PAGE); (4) articles written English. Alternatively, the exclusion criteria are (1) duplicate investigations, (2) investigations that do not have a list of analyzed proteins, and (3) nonexperimental investigations.

### 2.3. Bioinformatic Analysis

The list of proteins (Supplementary Table 1) of the studies selected to perform Genetic Ontology analysis (GO) was extracted including molecular function (MF), biological processes (BP), cellular components (CC), and pathway enrichment (KEGG) which were performed using the DAVID bioinformatics database (https://david.ncifcrf.gov/tools.jsp) to know the components and biological mechanisms affected in the Caco-2 cell line by the administration of AgNPs [14]. A protein-protein interaction network (PPI) was built on knowing the connectivity and understanding the biological phenomena involved in the administration of AgNPs. The analysis was performed using the interactive gene retrieval database (STRING) (http://string-db.org/) [15]. Additionally, the identified hub genes were analyzed in the UALCAN (http://ualcan.path.uab.edu/) and GEPIA2 (http://gepia2.cancer-pku.cn/#index) databases to know the degree of survival and gene expression for colon adenocarcinoma (COAD) and rectum adenocarcinoma (READ) [16, 17]. Finally, the database TISIDB (http://cis.hku.hk/TISIDB/search.php) was used to know the infiltration of immune cells in COAD and READ [18]. The cut-off points considered in the databases include *p* < 0.05, rho of Spearman > 0.300, and false discovery rate (FDR) < 0.05.

## 3. Results

### 3.1. Literature Selection and List Proteins

Table 1 shows the literature selected after applying the inclusion and exclusion criteria supplemented with information on the characteristics of the AgNPs, their production, the dose administered, and the number of proteins identified. The list of proteins was extracted from the selected articles for further analysis in the bioinformatics databases (Supplementary Table 1). The total number of spots with differential expression in the four studies was 616; 43 were not associated with any gene or protein. We use proteins expressed differentially in at least two experiments (47) to perform the subsequent analysis (Supplementary Table 2).

### 3.2. Enriched Analysis of Gene Ontology and Metabolic Pathways

We use the DAVID database to perform functional enrichment analysis of Gene Ontology and KEGG. The results obtained are shown in Table 2. The cut-off criterion established was FDR < 0.05. The analysis for BP showed enrichment for the terms cell-cell adhesion and gluconeogenesis. The CC analysis indicated that AgNPs affect the expression of proteins related to extracellular exosome, cytoplasm, and centriole. The MF results suggest deregulation in the proteins for cadherin binding involved in cell-cell adhesion and poly(A) RNA binding. Alternatively, the results obtained in the KEGG enrichment pathways suggest that the administration of AgNPs affects the pathways involved in carbon metabolism and the biosynthesis of amino acids.

### 3.3. Protein-Protein Interaction (PPI) Network

**Figure 2:**
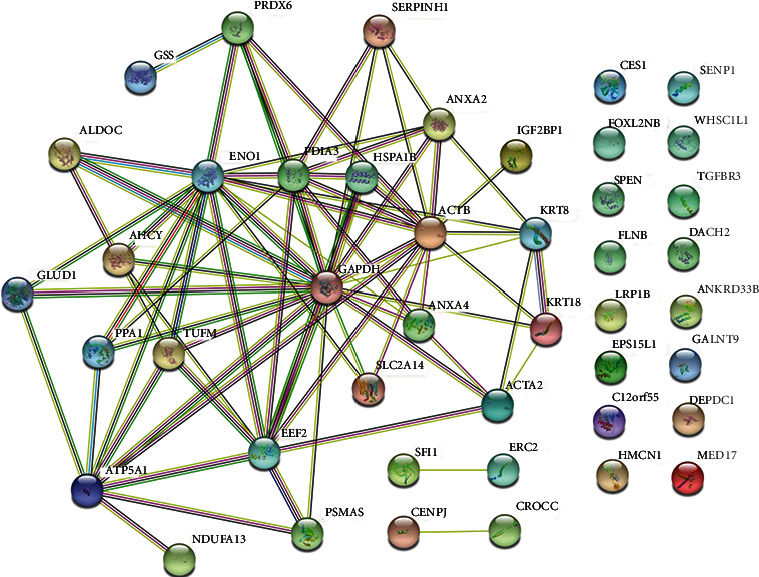
PPI network. The edges represent specific and significant protein-protein associations. Blue and purple borders represent known interactions from curated and experimentally determined databases, respectively. Predicted interactions of gene neighborhoods, gene fusion, and gene cooccurrence are identified in green, red, and navy blue, respectively. Other borders identified as grass green, black, and gray represent text mining, coexpression, and protein homology.

The STRING database helps us better form predicted functional associations of proteins to study the Caco-2 cell line. In Figure 2, we show the PPI network. We built a network with medium confidence (0.400). The interactome obtained consists of 75 edges, 44 nodes, and an average node degree of 3.41. The nodes with the highest grades were examined as hub genes.  Table 3 shows hub genes with at least ten interactions.

### 3.4. Analysis of Expression and Prognostic of the Hub Genes

**Figure 3:**
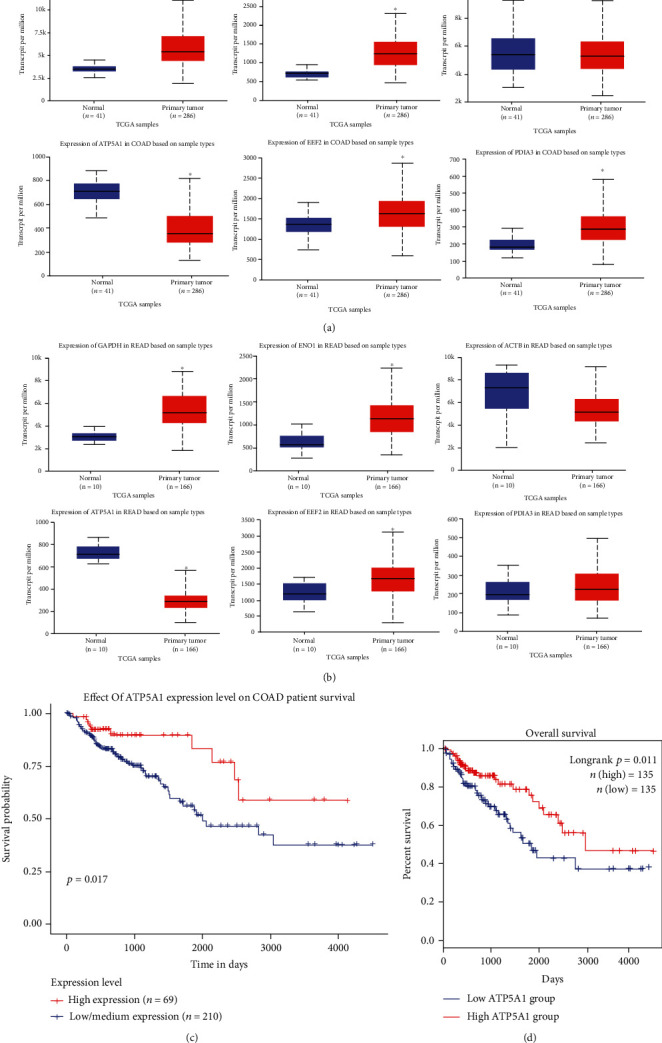
Expression of the hub genes in colon adenocarcinoma (COAD) (a), rectum adenocarcinoma (READ) (b), and survival analysis *ATP5A1* gene by UALCAN (c) and GEPIA2 (d) in COAD.

We use the UALCAN database to analyze the expression of the hub genes (shown in Table 3) in Genomics data from The Cancer Genome Atlas (TCGA) project. In this analysis, we divided colorectal cancer into two types: colon adenocarcinoma (COAD) and rectum adenocarcinoma (READ) (Figures 3(a) and 3(b)), respectively. The genes *GAPDH*, *ENO1*, *ATP5A1*, and *EEF2* showed differential expression in both COAD and READ (Figures 3(a) and 3(b)). The *PDIA3* gene was found to be overexpressed only in COAD (Figure 3(a)). Interestingly, the *ATP5A1* gene was only underexpressed in COAD and READ.

Additionally, the results of the analysis of survival curves performed in the UALCAN (Figure 3(c)) and GEPIA2 (Figure 3(d)) databases showed that the expression of the *ATP5A1* gene could be considered a prognostic factor. Low or medium expression of this gene was associated with a lower probability of survival in COAD. None of the other hub genes showed a relationship with colon survival in COAD and READ (results not shown). The analysis was conducted with the parameters established in the UALCAN and GEPIA2 databases.

### 3.5. Immune Cell Infiltration Analysis

We performed a correlation analysis between the expression of the hub genes (Table 3) and some immune cells including B, CD4+ T, CD8+ T, dendritic, and NK cells. This analysis was performed in both COAD and READ (Table 4). For this analysis, we use the TISIDB database. We take into account only correlations equal to or greater than 0.300. In COAD, we found significant positive correlations between the expression of the *ATP5A1* gene and the infiltration of CD4+ T cells (rho = 0.359, *p* = 2.36*e* − 15) and activated dendritic cells (rho = 0.330, *p* = 5.04*e* − 13). *ENO1* gene expression also positively correlated with CD4+ T cell infiltration (rho = 0.313, *p* = 8.25*e* − 12). In the analysis of READ, we observed negative correlations between the *GAPDH* gene and NK cells (rho = −0.309, *p* = 5.3*e* − 05) and between the *PDIA3* gene and CD4+ T cells (rho = 0.313, *p* = 4.11*e* − 05). We also observed some other correlations; however, they were weak, or the *p* was marginal. Spearman correlation plots between hub genes and immune cells for COAD and READ are shown in supplementary Figures 1 and 2, respectively.

## 4. Discussion

The high prevalence of cancer globally has led to the search for new therapeutic alternatives, including nanoparticles. Proteomics is a powerful technique for studying the effects of these nanoparticles on different types of cancer, including colorectal cancer. However, bioinformatics helps interpret the large amount of data obtained from proteomic studies. Therefore, the novelty of this research work is to integrate the available proteomic data on the effect of AgNPs administered in colorectal cancer cell lines through *in silico* analysis using different databases to learn more about their potential anticancer effect that support future therapeutic applications of AgNPs. Also, this work is the starting point for the results shown to be experimentally corroborated.

The results obtained from the Gene Ontology (Table 2) indicated that exposure to AgNPs affected the biological processes related to cell-cell adhesion and gluconeogenesis. Previous studies report that these two biological processes (BP) are actively involved in cancer development by inducing metastasis and energy regulation [19]. AgNPs also affect cellular components (CC), including extracellular exosome, cytoplasm, and centriole-related proteins. Centriole is a cell structure that is involved in cell cycle regulation, including colorectal cancer [20, 21]. Alternatively, the molecular function (MF) affected by exposure to AgNPs were cadherin and RNA binding, which plays an important role in the development of CRC [22, 23]. It is worth mentioning that some nanoparticles were functionalized, and the expression pattern of the cell line could be affected. In addition, we must take into account that cell apoptosis is another biological process that is induced by the administration of AgNPs according to what was reported by Abdellatif et al. [24].

Regarding the results of KEGG analysis on metabolic pathways affected by exposure to AgNPs (Table 2), the analysis suggests a dysregulation in carbon metabolism where gluconeogenesis and amino acid biosynthesis are involved. Carbon metabolism is necessary for synthesizing nucleotides and reducing cofactors and other components essential for the survival of cancer cells [19]. Therefore, these first results show that AgNPs affect proteins related to metabolic pathways and biological processes widely described in cancer. Consequently, an alteration in the genes related to this metabolic pathway due to the administration of AgNPs should be studied.

It is worth noting that the results obtained agree with a proteomic and metabolomic study that indicates that the exposure of gold nanoparticles (AuNPs) in Caco-2 cells produced an alteration in the mechanisms of cell-cell adhesion, integrity of the cytoskeleton, synthesis of amino acids, and alterations in cell growth and proliferation [25].

On the other hand, we identified hub genes affected by exposure to AgNPs on the Caco-2 cell line (Table 3). Then, we compared the expression of these hub genes in the UALCAN database. *GAPDH* was overexpressed in COAD and READ (Figure 3). Gioria et al. [26] reported that the GAPDH protein was downexpressed in the Caco-2 cell line by exposure for 24 h to 30 nm AgNPs at a concentration of 1 and 10 *μ*g/mL. According to the information reported by Tang et al. [27], inhibition of the GAPDH enzyme led to the death of cancer cells and prevented the development of metastases. Furthermore, GAPDH was suggested as a target for CRC treatment.

*ENO1* and *EEF2* are other hub genes identified that are overexpressed in COAD and READ according to the results obtained from the UALCAN database (Figure 3). Gioria et al. [26] reported that exposure to AgNPs at a concentration of 10 *μ*g/mL for 24 h showed a downward expression of *ENO1*. This finding is interesting because *ENO1* silencing inhibited the growth and migration of colon cancer cell lines in *in vitro* studies. Additionally, it is considered a therapeutic agent against CRC [28]. Alternatively, the hub gene *EEF2* was downexpressed after exposure to AgNPs at a concentration of 1 *μ*g/mL for 72 h [26]. According to Oji et al. [29], a low expression could limit the growth of cancer cells.

In the case of the *ATP5A1* gene, the UALCAN results showed a downward expression of the gene in COAD and READ. In agreement with Gioria et al. [26], the administration of AgNPs (1 and 10 *μ*g/mL for 24 h) causes overexpression of this gene, which would inhibit tumor development [30]. Likewise, the overexpression of this gene resulting from the administration of AgNPs could increase patient survival with CRC because other researchers have also reported that the downexpression of *ATP5A1* is associated with a low survival rate in colon cancer patients [31].

In this study, we analyzed the infiltration of immune cells using the TISIDB database. CRC is related to deficiencies in the immune system. Immune cells can vary phenotypically and functionally, creating an ideal immunological microenvironment for tumor inhibition or development [32]. The results indicate a positive regulation between the expression of the *ATP5A1* gene and the infiltration of CD4+ T cells as well as activated dendritic cells and a positive regulation between the expression of the *ENO1* gene and the CD4+ T cells in COAD (Table 4). This finding is interesting because high dendritic cell activity provides antitumor responses in CRC, and high CD4+ T cell densities have been associated with a better prognosis for CRC survival [33, 34]. Gioria et al. [26] observed that exposure to AgNPs (1 and 10 *μ*g/mL for 24 h) upregulated the protein ATP5A1. In the same way, they observed that the administration of AgNPs (1 *μ*g/mL for 24 h) increased the expression of *ENO1*. Therefore, the administration of nanoparticles enhances the activation of these cells and increases anticancer activity.

Alternatively, a negative correlation was observed between the *GAPDH* gene and NK cells on READ (Table 4). Gioria et al. [26] reported an overexpression of GAPDH by exposure to AgNPs at a concentration of 10 *μ*g/mL for 72 h. In accordance with what was suggested by Terren et al. [35], overexpression of this gene could inhibit interferon-*γ* (IFN-*γ*) production leading to increased cytotoxicity of NK cells. NK cells contribute activity against CRC, but experimental studies must be implemented to corroborate this result [36].

To conclude, we cannot fail to mention that the main challenge that must be overcome before thinking about the therapeutic use of AgNPs is the generation of toxic effects. In vivo studies evaluating the distribution and excretion of AgNPs have reported that AgNPs are distributed in vital organs including the liver, lungs, spleen, brain, heart, kidneys, testis, and thymus. The studies agreed that silver accumulation was higher in the liver and spleen, suggesting that they are target organs for the developing toxicity. Additionally, it was observed that the toxic effect depends on the particle size; the excretion was higher in feces than in urine, so AgNPs could have biliary activity and AgNPs have a different effect from that of ionic silver [37–39].

## 5. Perspectives

One of the critical limitations in conducting this bioinformatics study was that we did not generate independent lists of upregulated and downregulated proteins for analysis because the selected investigations were conducted under different conditions, including exposure times, concentrations, and physicochemical characteristics of the administered AgNPs. However, this study sets the tone for future experimental research to focus its efforts on the standardization of these conditions and evaluate the effect considering the findings found in this study.

Moreover, the cytotoxic effect has already been reviewed that can cause the administration of AgNPs [40]. However, *in vitro* and *in vivo* studies suggest that the toxicity of AgNPs depends on the administered dose, particle size, exposure time, and the coating present [41]. According to the literature, more studies are needed to know the biodistribution, accumulation, and viable routes of exposure in order to determine the toxicological threshold of AgNPs [42]. Future research should focus on the production of AgNPs by implementing green or biological synthesis methods. The benefit of these synthetic methods compared to chemical synthetic methods is that they could help mitigate the undesirable effects of the administration of AgNPs due to the generation of nanoparticles with high biocompatibility, less agglomeration, maximum clearance, and less toxicity, which could contribute to enhancing the therapeutic effect of AgNPs [43, 44].

## 6. Conclusion

The predictive analysis results indicated that the administration of AgNPs causes dysregulation of genes related to cell-cell adhesion, cytoplasm, centriole, and carbon metabolism, which contributes to anticancer activity. It was determined that the deregulation of hub genes *GAPDH*, *ENO1*, *EEF2*, and *ATP5A1* causes cellular apoptosis and antiproliferative and antimetastatic effects. Finally, hub genes and immune cell infiltration were related, finding a positive correlation between *ATP5A1* with CD4+ T and dendritic cells. Similarly, a positive correlation between the *ENO1* gene with CD4+ T cells was observed. Alternatively, a negative correlation between *GAPDH* and NK cells was discovered. These findings are fascinating since the administration of AgNPs impacts these genes and, in turn, the infiltration of cells of the immune system to promote the anticancer activity in CRC.

## Figures and Tables

**Table 1:** Selected literature.

Title	Nanoparticle size	Functionality	Synthesis method	Dosing	Proteins identified	Author and year
Proteomic responses of human intestinal Caco-2 cells exposed to silver nanoparticles and ionic silver	14 nm	Surfactant-coated stabilized with polyethylene glycol-25 (PEG-25) glyceryl trioleate (Tagat TO V) and polyethylene glycol-20 (PEG-20) sorbitan monolaurate (Tween20TM),	AgPURE™ from rent a scientist GmbH (Regensburg, Germany)	2.5 and 25 *μ*g/mL for 24 h	169	Oberemm et al., 2016 [45]
^∗^Effects of silver nanoparticles and ions on a coculture model for the gastrointestinal epithelium	20 and 200 nm	Not indicated	Were obtained from plasma-Chem GmbH (Berlin, Germany).	1 mg/L AgNPs-20, AgNPs-200 nm for 24 h	61	Georgantzopoulou et al., 2016 [46]
Protein corona analysis of silver nanoparticles links to their cellular effects	11.1 nm in aquarius stock and 21.3 nm in cell culture medium.	Surfactant-coated	AgPURE™ from rent a scientist GmbH (Regensburg, Germany).	25 *μ*g/mL for 24 h	54	Juling et al., 2017 [47]
Proteomics study of silver nanoparticles on Caco-2 cells	30 nm	Citrate stabilized	Reduction of AgNO3 with citrate and tannic acid	1 or 10 *μ*g/mL for 24 or 72 h	614	Gioria et al., 2018 [26]

^∗^The experiments of this research were carried out using cocultures (Caco-2/TC7 and HT29-MTX) so the results of proteomic studies are not only of the Caco-2 cell line.

**Table 2:** Functional enrichment analysis of deregulated proteins by exposure to AgNPs in Caco-2 cell.

Category	Term	Genes	*p* value	FDR
GOTERM_BP_DIRECT	Cell-cell adhesion	*ANXA2*, *ENO1*, *EPS15L1*, *EEF2*, *FLNB*, *HSPA1A*, *KRT18*, *PRDX6*	3.1*e*-06	9.6*e*-04
	Gluconeogenesis	*ALDOC*, *ENO1*, *GOT2*, *GAPDH*	1.5*e*-4	2.4*e*-2
GOTERM_CC_DIRECT	Extracellular exosome	*NDUFA13*, *TUFM*, *ACTB*, *ACTA2*, *AHCY*, *ALDOC*, *ANXA2*, *ANXA4*, *CROCC*, *ENO1*, *EEF2*, *FLNB*, *GOT2*, *GSS*, *GAPDH*, *HMCN1*, *KRT18*, *KRT8*, *PRDX6*, *PSMA5*, *PDIA3*, *PPA1*, *SERPINH1*, *SPEN*, *TGFBR3*	3.1*e*-10	3.5*e*-8
	Cytoplasm	*ERC2*, *NDUFA13*, *SENP1*, *ACTB*, *ACTA2*, *AHCY*, *ANXA4*, *CFAP54*, *CROCC*, *ENO1*, *EEF2*, *FLNB*, *GLUD1*, *GAPDH*, *HSPA1A*, *IGF2BP1*, *KRT18*, *KRT8*, *PRDX6*, *PSMA5*, *PPA1*, *TGFBR3*	1.9*e*-3	4.8*e*-2
	Centriole	*SFI1*, *CENPJ*, *CROCC*, *HSPA1A*	2.1*e*-3	4.8*e*-2
GOTERM_MF_DIRECT	Cadherin binding involved in cell-cell adhesion	*ANXA2*, *ENO1*, *EPS15L1*, *EEF2*, *FLNB*, *HSPA1A*, *KRT18*, *PRDX6*	4.7*e*-6	6.5*e*-4
	Poly(A) RNA binding	*TUFM*, *ANXA2*, *ENO1*, *EEF2*, *FLNB*, *GOT2*, *IGF2BP1*, *KRT18*, *PDIA3*, *SERPINH1*, *SPEN*	2.3*e*-4	1.6*e*-2
KEGG_PATHWAY	Carbon metabolism	*ALDOC*, *ENO1*, *GLUD1*, *GOT2*, *GAPDH*	3.3*e*-4	2.1*e*-2
	Biosynthesis of amino acids	*ALDOC*, *ENO1*, *GOT2*, *GAPDH*	1.3*e*-3	3.3*e*-2
	Metabolic pathways	*NDUFA13*, *AHCY*, *ALDOC*, *CES1*, *ENO1*, *GLUD1*, *GOT2*, *GSS*, *GAPDH*, *PRDX6*, *GALNT9*	3.3*e*-2	3.3*e*-2

**Table 3:** Hub genes with at least ten interactions involved in the effect of AgNPs on the Caco-2 cell line.

Gene symbol	Protein	Function	Degree
*GAPDH*	Glyceraldehyde-3-phosphate dehydrogenase	This enzyme plays an important role in glycolysis and nuclear functions.	21
*ENO1*	Alpha-enolase	An enzyme that plays an important role in glycolysis, growth control, tolerance to hypoxia, and allergic responses.	17
*ACTB*	Actin, cytoplasmic 1	Conserved protein that produces filaments that form networks in the cytoplasm of cells.	15
*ATP5A1*	ATP synthase subunit alpha, mitochondrial	Enzyme that produces ATP from ADP in the presence of a proton gradient that is generated by electron transport complexes in the respiratory chain.	11
*EEF2*	Elongation factor 2	It plays an important role in ribosomal translocation in the elongation of translation.	10
*PDIA3*	Protein disulfide-isomerase A3	Participates in the rearrangement of disulfide bonds in proteins.	10

**Table 4:** Correlation analysis between hub genes and immune cells on COAD and READ.

Hub genes	B cell	CD4 cell	CD8 cell	Dendritic cell	NK cell
COAD					
*GAPDH*	*-0.198* **2.05** **e** ^**-05**^	*0.147* **0.00166**	*0.133* **0.015**	*0.203* **1.27** **e** ^**-05**^	*-0.196* **2.36** **e** ^**-05**^
*ENO1*	*-0.174* **0.000182**	*0.313* **8.25** **e** ^**-12**^	*0.203* **1.21** **e** ^**-05**^	*0.248* **8.3** **e** ^**-08**^	*-0.068* **0.145**
*ACTB*	*-0.024* **0.609**	*-0.084* **0.0707**	*0.089* **0.0578**	*0.253* **4.15** **e** ^**-08**^	*0.048* **0.304**
*ATP5A1*	*-0.025* **0.594**	*0.359* **2.36** **e** ^**-15**^	*0.229* **7.25** **e** ^**-07**^	*0.330* **5.04** **e** ^**-13**^	*-0.043* **0.356**
*EEF2*	*-0.127* **0.00641**	*-0.167* **0.000338**	*-0.114* **0.0147**	*-0.117* **0.012**	*-0.146* **0.00179**
*PDIA3*	*-0.211* **5.32** **e** ^**-06**^	*0.291* **2.65** **e** ^**-10**^	*0.058* **0.213**	*-0.003* **0.951**	*-0.091* **0.0504**
READ					
*GAPDH*	*-0.103* **0.185**	*-0.068* **0.384**	*0.088* **0.256**	*0.092* **0.239**	*-0.309* **5.3** **e** ^**-05**^
*ENO1*	*-0.282* **0.000231**	*0.122* **0.116**	*-0.047* **0.549**	*0.082* **0.291**	*-0.297* **0.000105**
*ACTB*	*-0.063* **0.415**	*-0.285* **0.000196**	*-0.06* **0.441**	*0.147* **0.0573**	*0.017* **0.825**
*ATP5A1*	*-0.037* **0.631**	*0.167* **0.0308**	*0.128* **0.099**	*0.254* **0.000967**	*-0.139* **0.0736**
*EEF2*	*0.034* **0.658**	-*0.253* **0.000998**	*0.066* **0.399**	*-0.022* **0.775**	*-0.122* **0.116**
*PDIA3*	*-0.172* **0.0265**	*0.313* **4.11** **e** ^**-05**^	*-0.099* **0.202**	*-0.036* **0.643**	*-0.032* **0.683**

rho value; **pvalue**.

## Data Availability

The data that supports the findings of this study are available in the supplementary material of this article.

## Supplementary Materials

Supplementary Materials The supplementary material contains the list of proteins obtained from the research work used in this work. Likewise, correlation plots between hub genes and immune cells for COAD and READ are shown in supplementary Figures 1 and 2, respectively.
