# Supplementary material for: Bioinformatic Analysis of the Effect of Silver Nanoparticles on Colorectal Cancer Cell Line
Source: Biomed Res Int. 2022 Apr 11;2022:6828837. doi: 10.1155/2022/6828837 (PMC9015850; doi:10.1155/2022/6828837)
Supplement: Supplementary Materials — The supplementary material contains the list of proteins obtained from the research work used in this work. Likewise, correlation plots between hub genes and immune cells for COAD and READ are shown in supplementary Figures 1 and 2, respectively. [file 6828837.f1.zip › Supplementary tables 1 (1).docx]

**Supplementary Table 1.** List of proteins obtained from selected research.

| **Description** | **UniProt ID** | **Gen** |
| --- | --- | --- |
| 17 beta-hydroxysteroid dehydrogenase | Q13034 | *17BHSDI* |
| Alpha-2-macroglobulin-like protein 1 | A8K2U0 | *A2ML1* |
| Multidrug resistance-associated protein 5 | O15440 | *ABCC5* |
| ATP-binding cassette sub-family F member 1 | Q8NE71 | *ABCF1* |
| Long-chain-fay-acid--CoA ligase ACSBG2 | Q5FVE4 | *ACSBG2* |
| Acyl-coenzyme A synthetase ACSM1, mitochondrial | Q08AH1 | *ACSM1* |
| Actin, aortic smooth muscle | P62736 | *ACTA2* |
| Actin, cytoplasmic 1 | P60709 | *ACTB* |
| Disintegrin and metalloproteinase domain-containing protein 28 (Fragment) | H0YBQ8 | *ADAM28* |
| Adhesion G-protein coupled receptor G2 | Q8IZP9 | *ADGRG2* |
| AF4/FMR2 family member 1 | P51825 | *AFF1* |
| Aftiphilin | Q6ULP2 | *AFTPH* |
| Adenosylhomocysteinase | P23526 | *AHCY* |
| Retinal dehydrogenase 1 | P00352 | *ALDH1A1* |
| Aldehyde dehydrogenase, mitochondrial | P05091 | *ALDH2* |
| Alsin | Q96Q42 | *ALS2* |
| Angiomon-like protein 1 | Q8IY63 | *AMOTL1* |
| Ankyrin-1 | P16157 | *ANK1* |
| Ankyrin-3 | Q12955 | *ANK3* |
| Putative ankyrin repeat domain-containing protein 26-like protein | Q6NSI1 | *ANKRD26P1* |
| Ankyrin repeat domain-containing protein 33B | A6NCL7 | *ANKRD33B* |
| Ankyrin repeat and SAM domain-containing protein 4B | Q8N8V4 | *ANKS4B* |
| Protein ANKUB1 | A6NFN9 | *ANKUB1* |
| Anoctamin-1 | Q5XXA6 | *ANO1* |
| Isoform 2 of Annexin A11 | P50995 | *ANXA11* |
| Annexin A2 | P07355 | *ANXA2* |
| Annexin A4 | P09525 | *ANXA4* |
| Annexin A5 | P08758 | *ANXA5* |
| AP-1 complex subunit gamma-1 (Fragment) | H3BR36 | *AP1G1* |
| AP-3 complex subunit beta-2 | Q13367 | *AP3B2* |
| Arf-GAP with Rho-GAP domain, ANK repeat and PH domain-containing protein 3 | Q8WWN8 | *ARAP3* |
| Rho GTPase-activating protein 18 | Q8N392 | *ARHGAP18* |
| Rho GTPase-acvang protein 21 | Q5T5U3 | *ARHGAP21* |
| Rho guanine nucleode exchange factor 10 | O15013 | *ARHGEF10* |
| Rho guanine nucleode exchange factor 6 | Q15052 | *ARHGEF6* |
| AT-rich interacve domain-containing protein 4A (Fragment) | H7C485 | *ARID4A* |
| AT-rich interactive domain-containing protein 5B | Q14865 | *ARID5B* |
| Armadillo repeat-containing X-linked protein 4 | Q5H9R4 | *ARMCX4* |
| Actin-related protein 2/3 complex subunit 5 | O15511 | *ARPC5* |
| Ankyrin repeat and SOCS box protein 15 | Q8WXK1 | *ASB15* |
| Ankyrin repeat and SOCS box protein 16 | Q96NS5 | *ASB16* |
| Putative Polycomb group protein ASXL2 | Q76L83 | *ASXL2* |
| Putative Polycomb group protein ASXL3 | Q9C0F0 | *ASXL3* |
| Ankyrin repeat, SAM and basic leucine zipper domain-containing protein 1 | Q8WWH4 | *ASZ1* |
| Serine-protein kinase ATM | Q13315 | *ATM* |
| Endoplasmic reticulum transmembrane helix translocase | Q9HD20 | *ATP13A1* |
| Plasma membrane calcium-transporting ATPase 1 | P20020 | *ATP2B1* |
| ATP synthase subunit alpha, mitochondrial | P25705 | *ATP5F1A* |
| Transcription regulator protein BACH1 | O14867 | *BACH1* |
| Brain-specific angiogenesis inhibitor 1-associated protein 2-like protein 1 92833 | Q9UHR4 | *BAIAP2L1* |
| Bromodomain adjacent to zinc finger domain protein 2A | Q9UIF9 | *BAZ2A* |
| Breast carcinoma-amplified sequence 3 | Q9H6U6 | *BCAS3* |
| Class E basic helix-loop-helix protein 40 | O14503 | *BHLHE40* |
| Uncharacterized protein C6orf222 | P0C671 | *BNIP5* |
| Brother of CDO | Q9BWV1 | *BOC* |
| BRI3-binding protein | Q8WY22 | *BRI3BP* |
| Bromodomain and WD repeat-containing protein 1 | Q9NSI6 | *BRWD1* |
| Uncharacterized protein C17orf100 | A8MU93 | *C17orf100* |
| Uncharacterized protein C20orf96 | Q9NUD7 | *C20orf96* |
| Uncharacterized protein C2orf47, mitochondrial | Q8WWC4 | *C2orf47* |
| Complement C3 | P01024 | *C3* |
| Voltage-dependent P/Q-type calcium channel subunit alpha-1A | O00555 | *CACNA1A* |
| Voltage-dependent L-type calcium channel subunit alpha-1C | Q13936 | *CACNA1C* |
| Voltage-dependent R-type calcium channel subunit alpha-1E | Q15878 | *CACNA1E* |
| Calretinin | P22676 | *CALB2* |
| Calreticulin | P27797 | *CALR* |
| Calumenin | O43852 | *CALU* |
| Leucine-rich repeat-containing protein 16C | Q6F5E8 | *CARMIL2* |
| Protein CBFA2T3 | O75081 | *CBFA2T3* |
| Coiled-coil domain-containing protein 136 | Q96JN2 | *CCDC136* |
| Coiled-coil domain-containing protein 142 | Q17RM4 | *CCDC142* |
| Coiled-coil domain-containing protein 15 | Q0P6D6 | *CCDC15* |
| Coiled-coil domain-containing protein 150 | Q8NCX0 | *CCDC150* |
| Coiled-coil domain-containing protein 170 | Q8IYT3 | *CCDC170* |
| Coiled-coil domain-containing protein 171 | Q6TFL3 | *CCDC171* |
| Coiled-coil domain-containing protein 6 | Q16204 | *CCDC6* |
| Coiled-coil domain-containing protein 71L | Q8N9Z2 | *CCDC71L* |
| Coiled-coil domain-containing protein 73 | Q6ZRK6 | *CCDC73* |
| Coiled-coil domain-containing protein 74A | Q96AQ1 | *CCDC74A* |
| Protein Daple | Q9P219 | *CCDC88C* |
| Cyclin-F | P41002 | *CCNF* |
| Cyclin-L1 | Q9UK58 | *CCNL1* |
| T-complex protein 1 subunit theta | P50990 | *CCT8* |
| Serine/threonine-protein kinase MRCK gamma 663 | Q6DT37 | *CDC42BPG* |
| Cadherin-17 | Q12864 | *CDH17* |
| Carcinoembryonic antigen-related cell adhesion molecule 1 | P13688 | *CEACAM1* |
| CCAAT/enhancer-binding protein zeta | Q03701 | *CEBPZ* |
| Cadherin EGF LAG seven-pass G-type receptor 2 | Q9HCU4 | *CELSR2* |
| Centromere protein J | Q9HC77 | *CENPJ* |
| Centrosomal protein of 152 kDa | O94986 | *CEP152* |
| Centrosomal protein of 70 kDa | Q8NHQ1 | *CEP70* |
| Liver carboxylesterase 1 | P23141 | *CES1* |
| Cilia- and flagella-associated protein 54 | Q96N23 | *CFAP54* |
| Cilia- and flagella-associated protein 70 | Q5T0N1 | *CFAP70* |
| Cofilin 1 (Non-muscle), isoform CRA_a | G3V1A4 | *CFL1* |
| Chromodomain-helicase-DNA-binding protein 2 | O14647 | *CHD2* |
| Neural cell adhesion molecule L1-like protein | O00533 | *CHL1* |
| Charged multivesicular body protein 4b | Q9H444 | *CHMP4B* |
| Chondroitin sulfate glucuronyltransferase | Q9P2E5 | *CHPF2* |
| Acetylcholine receptor subunit epsilon | Q04844 | *CHRNE* |
| Protein capicua homolog | I3L2J0 | *CIC* |
| Cell death activator CIDE-B | Q9UHD4 | *CIDEB* |
| Creatine kinase B-type | P12277 | *CKB* |
| Calcium-activated chloride channel regulator family member 3 | Q9Y6N3 | *CLCA3P* |
| H(+)/Cl(-) exchange transporter 5 | P51795 | *CLCN5* |
| Chloride intracellular channel protein 1 | O00299 | *CLIC1* |
| CAP-Gly domain-containing linker protein 4 | Q8N3C7 | *CLIP4* |
| ATP-dependent Clp protease ATP-binding subunit clpX-like, mitochondrial | O76031 | *CLPX* |
| UMP-CMP kinase | P30085 | *CMPK1* |
| Contactin-associated protein-like 2 | Q9UHC6 | *CNTNAP2* |
| Contactin-associated protein-like 3B | Q96NU0 | *CNTNAP3B* |
| Collagen alpha-2(XI) chain | P13942 | *COL11A2* |
| Collagen alpha-1(XIX) chain | Q14993 | *COL19A1* |
| Collagen alpha-1(XXVII) chain | Q8IZC6 | *COL27A1* |
| Collagen alpha-5(VI) chain | A8TX70 | *COL6A5* |
| Collagen alpha-2(IX) chain | Q14055 | *COL9A2* |
| CPX chromosomal region candidate gene 1 protein | Q8N123 | *CPXCR1* |
| Probable carboxypepdase X1 | Q96SM3 | *CPXM1* |
| Rootletin | Q5TZA2 | *CROCC* |
| Cartilage-associated protein | O75718 | *CRTAP* |
| Very large A-kinase anchor protein | Q68DQ2 | *CRYBG3* |
| Citrate synthase | A0A0C4DGI3 | *CS* |
| Granulocyte colony-stimulating factor receptor | Q99062 | *CSF3R* |
| Isoform 3 of Casein kinase I isoform alpha | P48729 | *CSNK1A1* |
| Centrosome and spindle pole-associated protein 1 | Q1MSJ5 | *CSPP1* |
| C-terminal-binding protein 1 | Q13363 | *CTBP1* |
| Src substrate cortactin | Q14247 | *CTTN* |
| Cullin-1 | Q13616 | *CUL1* |
| Cullin-5 | Q93034 | *CUL5* |
| Cytochrome b5 | P00167 | *CYB5A* |
| Cylicin-2 | Q14093 | *CYLC2* |
| Cytochrome P450 2D6 | P10635 | *CYP2D6* |
| Disheveled-associated activator of morphogenesis 1 | Q9Y4D1 | *DAAM1* |
| Disheveled-associated activator of morphogenesis 2 | Q86T65 | *DAAM2* |
| Dachshund homolog 2 | Q96NX9 | *DACH2* |
| D-amino-acid oxidase | P14920 | *DAO* |
| Acyl-CoA-binding protein | P07108 | *DBI* |
| DDB1- and CUL4-associated factor 4-like protein 1 | Q3SXM0 | *DCAF4L1* |
| DNA damage-binding protein 1 | Q16531 | *DDB1* |
| Nucleolar RNA helicase 2 | Q9NR30 | *DDX21* |
| Probable ATP-dependent RNA helicase DDX23 | Q9BUQ8 | *DDX23* |
| Probable ATP-dependent RNA helicase DDX31 | Q9H8H2 | *DDX31* |
| ATP-dependent RNA helicase DDX39A | O00148 | *DDX39A* |
| Probable ATP-dependent RNA helicase DDX46 | Q7L014 | *DDX46* |
| Isoform 2 of DENN domain-containing protein 2C | Q68D51 | *DENND2C* |
| DENN domain-containing protein 3 | A2RUS2 | *DENND3* |
| DEP domain-containing protein 1A | Q5TB30 | *DEPDC1* |
| UPF0317 protein C14orf159, mitochondrial | Q7Z3D6 | *DGLUCY* |
| ATP-dependent RNA helicase DHX29 | Q7Z478 | *DHX29* |
| Protein diaphanous homolog 3 | Q9NSV4 | *DIAPH3* |
| Disco-interacting protein 2 homolog B | Q9P265 | *DIP2B* |
| Phosphoinoside phospholipase C (Fragment) | Q9UFY1 | *DKFZp434N101* |
| Putative uncharacterized protein DKFZp686P17171 | Q63HR1 | *DKFZp686P17171* |
| Dystrophin | P11532 | *DMD* |
| Dynein assembly factor 1, axonemal | Q8NEP3 | *DNAAF1* |
| Dynein heavy chain 10, axonemal | Q8IVF4 | *DNAH10* |
| Dynein heavy chain 11, axonemal | Q96DT5 | *DNAH11* |
| Dynein heavy chain 14, axonemal | Q0VDD8 | *DNAH14* |
| Dynein heavy chain 3, axonemal | Q8TD57 | *DNAH3* |
| DnaJ homolog subfamily A member 4 | Q8WW22 | *DNAJA4* |
| DnaJ homolog subfamily C member 10 | Q8IXB1 | *DNAJC10* |
| DnaJ homolog subfamily C member 15 | Q9Y5T4 | *DNAJC15* |
| Dynamin-1 | Q05193 | *DNM1* |
| DNA (cytosine-5)-methyltransferase 1 | P26358 | *DNMT1* |
| Dedicator of cytokinesis protein 7 | Q96N67 | *DOCK7* |
| Docking protein 4 (Fragment) | H3BVB4 | *DOK4* |
| Isoform 2 of Protein Dok-7 | Q18PE1 | *DOK7* |
| Dihydropyrimidine dehydrogenase [NADP(+)] | Q12882 | *DPYD* |
| Dihydropyrimidinase-related protein 2 | Q16555 | *DPYSL2* |
| Dystrophin-related protein 2 | Q13474 | *DRP2* |
| Desmoglein-2 | Q14126 | *DSG2* |
| Dystonin | Q03001 | *DST* |
| E3 ubiquitin-protein ligase DTX3L | Q8TDB6 | *DTX3L* |
| WD repeat-containing protein 60 | Q8WVS4 | *DYNC2I1* |
| Dual specificity tyrosine-phosphorylation-regulated kinase 2 | Q92630 | *DYRK2* |
| Transcription factor E2F7 | Q96AV8 | *E2F7* |
| Enoyl-CoA hydratase, mitochondrial | P30084 | *ECHS1* |
| Isoform 2 of Enoyl-CoA delta isomerase 1, mitochondrial | P42126 | *ECI1* |
| Enhancer of mRNA-decapping protein 4 | Q6P2E9 | *EDC4* |
| Elongation factor 2 | P13639 | *EEF2* |
| Eukaryotic elongation factor 2 kinase | O00418 | *EEF2K* |
| EF-hand domain-containing family member B | Q8N7U6 | *EFHB* |
| Eukaryotic translation initiation factor 4 gamma 3 | O43432 | *EIF4G3* |
| RNA polymerase II elongation factor ELL2 | O00472 | *ELL2* |
| Endonuclease G, mitochondrial | Q14249 | *ENDOG* |
| Cytosolic endo-beta-N-acetylglucosaminidase | Q8NFI3 | *ENGASE* |
| Alpha-enolase | P06733 | *ENO1* |
| Ectonucleotide pyrophosphatase/phosphodiesterase family member 5 | Q9UJA9 | *ENPP5* |
| Band 4.1-like protein 2 | E9PHY5 | *EPB41L2* |
| Epoxide hydrolase 1 | P07099 | *EPHX1* |
| Epidermal growth factor receptor substrate 15-like 1 | Q9UBC2 | *EPS15L1* |
| Receptor tyrosine-protein kinase erbB-3 | P21860 | *ERBB3* |
| ERC protein 2 | O15083 | *ERC2* |
| Extended synaptotagmin-2 | A0FGR8 | *ESYT2* |
| Electron transfer flavoprotein subunit alpha, mitochondrial (Fragment) | H0YLU7 | *ETFA* |
| Exocyst complex component 6B | Q9Y2D4 | *EXOC6B* |
| Prothrombin | P00734 | *F2* |
| 5'-nucleodase domain-containing protein 2 | NT5DC2 | *F8WEY1* |
| Fatty acid binding protein | P07148 | *FABP1* |
| Protein FAM135A | Q9P2D6 | *FAM135A* |
| Protein FAM184A | Q8NB25 | *FAM184A* |
| Protein FAM210A (Fragment) | K7EK00 | *FAM210A* |
| Protocadherin Fat 3 | Q8TDW7 | *FAT3* |
| Isoform 4 of Fc receptor-like B | Q6BAA4 | *FCRLB* |
| Fez family zinc finger protein 2 | Q8TBJ5 | *FEZF2* |
| Isoform Cytoplasmic of Fumarate hydratase, mitochondrial | P07954 | *FH* |
| FH2 domain-containing protein 1 | Q9C0D6 | *FHDC1* |
| Filamin-A-interacting protein 1 | Q7Z7B0 | *FILIP1* |
| Pepdyl-prolyl cis-trans isomerase FKBP4 | Q02790 | *FKBP4* |
| FLJ44955 protein | Q0VFX3 | *FLI44955* |
| Protein flightless-1 homolog | Q13045 | *FLII* |
| Filamin-B | O75369 | *FLNB* |
| Flollin-1 | O75955 | *FLOT1* |
| FOXL2 neighbor protein | Q6ZUU3 | *FOXL2NB* |
| Forkhead box protein Q1 | Q9C009 | *FOXQ1* |
| Fucose-1-phosphate guanylyltransferase | O14772 | *FPGT* |
| Tyrosine-protein kinase Fyn | P06241 | *FYN* |
| Polypeptide N-acetylgalactosaminyltransferase 10 | Q86SR1 | *GALNT10* |
| Polypeptide N-acetylgalactosaminyltransferase 9 | Q9HCQ5 | *GALNT9* |
| Polypeptide N-acetylgalactosaminyltransferase 6 | Q49A17 | *GALNTL6* |
| Glyceraldehyde-3-phosphate dehydrogenase | P04406 | *GAPDH* |
| Golgi-specific brefeldin A-resistance guanine nucleotide exchange factor 1 | Q92538 | *GBF1* |
| GRIP and coiled-coil domain-containing protein 2 | Q8IWJ2 | *GCC2* |
| Translational activator GCN1 | Q92616 | *GCN1* |
| N-acetyllactosaminide beta-1,6-N-acetylglucosaminyl-transferase, isoform C | Q8NFS9 | *GCNT2* |
| Glial fibrillary acidic protein (Fragment) | K7EJU1 | *GFAP* |
| Isoform 3 of Apoptosis-inducing factor 1, mitochondrial | O95831 | *GFIA1* |
| Mitochondrial elongation factor G | E5KND7 | *GFM1* |
| Glutamine--fructose-6-phosphate aminotransferase [isomerizing] 1 | Q06210 | *GFPT1* |
| Growth hormone variant | P01242 | *GH2* |
| Golgi-associated plant pathogenesis-related protein 1 | Q9H4G4 | *GLIPR2* |
| Glutaredoxin-3 | O76003 | *GLRX3* |
| Glutamate dehydrogenase 1, mitochondrial | P00367 | *GLUD1* |
| Golgin subfamily A member 2 | Q08379 | *GOLGA2* |
| Golgin subfamily A member 3 | Q08378 | *GOLGA3* |
| Aspartate aminotransferase, cytoplasmic | P17174 | *GOT1* |
| Aspartate aminotransferase | A0A024R6W0 | *GOT2* |
| Glycerol-3-phosphate dehydrogenase, mitochondrial | P43304 | *GPD2* |
| G patch domain and KOW motifs-containing protein | Q92917 | *GPKOW* |
| G-protein coupled receptor 4 | P46093 | *GPR4* |
| G-protein-signaling modulator 1 | Q86YR5 | *GPSM1* |
| GREB1-like protein | Q9C091 | *GREB1L* |
| Glutamate receptor ionotropic, kainate 4 | Q16099 | *GRIK4* |
| Glutamate receptor ionotropic, NMDA 1 | Q05586 | *GRIN1* |
| Gelsolin isoform a precursor | P06396 | *GSN* |
| Glutathione synthetase | P48637 | *GSS* |
| Glutathione S-transferase C-terminal domain-containing protein | Q8NEC7 | *GSTCD* |
| Glutathione S-transferase P | P09211 | *GSTP1* |
| General transcription factor 3C polypeptide 1 | Q12789 | *GTF3C1* |
| Nucleolar GTP-binding protein 1 | Q9BZE4 | *GTPBP4* |
| PTB domain-containing engulfment adapter protein 1 | Q9UBP9 | *GULP1* |
| Hyaluronan-binding protein 2 | Q14520 | *HABP2* |
| HCF N-terminal chain 5 | P51610 | *HCFC1* |
| Histone deacetylase 9 | Q9UKV0 | *HDAC9* |
| Hepatoma-derived growth factor-related protein 2 | Q7Z4V5 | *HDGFL2* |
| Heme-binding protein 2 | Q9Y5Z4 | *HEBP2* |
| Epididymis luminal protein 109 | V9HVX8 | *HEL-109* |
| Histidine triad nucleotide-binding protein 1 | P49773 | *HINT1* |
| Hemicentin-1 | Q96RW7 | *HMCN1* |
| Hemicentin-2 | Q8NDA2 | *HMCN2* |
| Heterogeneous nuclear ribonucleoprotein A1-like 2 | Q32P51 | *HNRNPA1L2* |
| 15-hydroxyprostaglandin dehydrogenase [NAD(+)] | P15428 | *HPGD* |
| Lysine-specific demethylase hairless | O43593 | *HR* |
| Hornerin | Q86YZ3 | *HRNR* |
| Heat shock 70 kDa protein 1A | P0DMV8 | *HSPA1A* |
| Heat shock 70 kDa protein 1-like | P34931 | *HSPA1L* |
| 78 kDa glucose-regulated protein | P11021 | *HSPA5* |
| 60 kDa heat shock protein, mitochondrial | P10809 | *HSPD1* |
| Mitochondrial heat shock 60kD protein 1 variant 1 | B3GQS7 | *HSPD1* |
| Isoform Beta of Heat shock protein 105 kDa | Q92598 | *HSPH1* |
| Serine protease HTRA3 | P83110 | *HTRA3* |
| Hypoxia up-regulated protein 1 | A0A087X054 | *HYOU1* |
| Isocitrate dehydrogenase [NADP] cytoplasmic | O75874 | *IDH1* |
| Isocitrate dehydrogenase 1 (Fragment) | Q0QER2 | *IDH1* |
| Interferon-induced protein with tetratricopeptide repeats 1B | Q5T764 | *IFIT1B* |
| Interferon-induced protein with tetratricopeptide repeats 5 | Q13325 | *IFIT5* |
| Isoform 3 of Intraflagellar transport protein 88 homolog | Q13099 | *IFT88* |
| Insulin-like growth factor 2 mRNA-binding protein 1 | Q9NZI8 | *IGF2BP1* |
| Insulin-like growth factor 2 mRNA-binding protein 2 | F8W930 | *IGF2BP2* |
| Immunoglobulin superfamily member 3 | O75054 | *IGSF3* |
| Interleukin-1 receptor-like 2 | Q9HB29 | *IL1RL2* |
| Type I inositol 1,4,5-trisphosphate 5-phosphatase | Q14642 | *INPP5A* |
| Protein asunder homolog | Q9NVM9 | *INTS13* |
| IQ and AAA domain-containing protein 1-like | A6NCM1 | *IQCA1L* |
| IQ domain-containing protein E | Q6IPM2 | *IQCE* |
| IQ motif and SEC7 domain-containing protein 1 | Q6DN90 | *IQSEC1* |
| Iron-responsive element-binding protein 2 | P48200 | *IREB2* |
| Intersecn-2 | Q9NZM3 | *ITSN2* |
| Janus kinase and microtubule-interacting protein 1 | Q96N16 | *JAKMIP1* |
| Protein Jumonji | Q92833 | *JARID2* |
| Probable JmjC domain-containing histone demethylation protein 2C | Q15652 | *JMJD1C* |
| KN motif and ankyrin repeat domain-containing protein 3 | Q6NY19 | *KANK3* |
| Potassium voltage-gated channel subfamily H member 6 | Q9H252 | *KCNH6* |
| Kielin/chordin-like protein | Q6ZWJ8 | *KCP* |
| BTB/POZ domain-containing protein KCTD16 | Q68DU8 | *KCTD16* |
| Far upstream element-binding protein 2 | A0A087WTP3 | *KHSRP* |
| Uncharacterized protein KIAA1107 | Q9UPP5 | *KIAA1107* |
| Uncharacterized protein KIAA1210 | Q9ULL0 | *KIAA1210* |
| Sickle tail protein homolog | Q5T5P2 | *KIAA1217* |
| Kinesin-like protein KIF16B | Q96L93 | *KIF16B* |
| Plasma kallikrein (Fragment) | H0YAC1 | *KLKB1* |
| Histone-lysine N-methyltransferase 2A | Q03164 | *KMT2A* |
| N-lysine methyltransferase SETD8 | Q9NQR1 | *KMT5A* |
| Keratin, type I cytoskeletal 18 | P05783 | *KRT18* |
| Keratin, type I cuticular Ha5 | Q92764 | *KRT35* |
| Keratin, type I cuticular Ha6 | O76013 | *KRT36* |
| Keratin, type II cytoskeletal 4 | P19013 | *KRT4* |
| Keratin, type II cytoskeletal 1b | Q7Z794 | *KRT77* |
| Keratin, type II cytoskeletal 8 | P05787 | *KRT8* |
| Keratin, type I cytoskeletal 9 | P35527 | *KRT9* |
| LINE-1 type transposase domain-containing protein 1 | Q5T7N2 | *L1TD1* |
| Laminin subunit gamma-1 | P11047 | *LAMC1* |
| Laminin subunit gamma-3 | Q9Y6N6 | *LAMC3* |
| Glycosyltransferase-like protein LARGE1 | O95461 | *LARGE1* |
| La-related protein 1 | Q6PKG0 | *LARP1* |
| La-related protein 1B | Q659C4 | *LARP1B* |
| L-lactate dehydrogenase B chain | P07195 | *LDHB* |
| Leiomodin-1 | P29536 | *LMOD1* |
| Low-density lipoprotein receptor-related protein 1B | Q9NZR2 | *LRP1B* |
| Low-density lipoprotein receptor-related protein 2 | P98164 | *LRP2* |
| Low-density lipoprotein receptor-related protein 5 | O75197 | *LRP5* |
| Low-density lipoprotein receptor-related protein 6 | O75581 | *LRP6* |
| Leucine-rich repeat serine/threonine-protein kinase 2 | Q5S007 | *LRRK2* |
| Leukotriene A-4 hydrolase | P09960 | *LTA4H* |
| Lymphocyte antigen 75 | O60449 | *LY75* |
| Lysosomal-trafficking regulator | Q99698 | *LYST* |
| MAP kinase-activating death domain protein | Q8WXG6 | *MADD* |
| Melanoma-associated antigen B4 | O15481 | *MAGEB4* |
| Membrane-associated guanylate kinase, WW and PDZ domain-containing protein 3 | Q5TCQ9 | *MAGI3* |
| Mannosyl-oligosaccharide 1,2-alpha-mannosidase IA | P33908 | *MAN1A1* |
| Mitogen-activated protein kinase kinase kinase 20 | Q9NYL2 | *MAP3K20* |
| MAP7 domain-containing protein 2 | Q96T17 | *MAP7D2* |
| Mitogen-activated protein kinase-binding protein 1 | O60336 | *MAPKBP1* |
| Microtubule-associated serine/threonine-protein kinase 4 | O15021 | *MAST4* |
| Methyl-CpG-binding domain protein 5 | Q9P267 | *MBD5* |
| Methylcrotonoyl-CoA carboxylase beta chain, mitochondrial | Q9HCC0 | *MCCC2* |
| Germinal-center associated nuclear protein | O60318 | *MCM3AP* |
| DNA replication licensing factor MCM4 | P33991 | *MCM4* |
| Malate dehydrogenase, cytoplasmic | P40925 | *MDH1* |
| Mediator of RNA polymerase II transcription subunit 17 | Q9NVC6 | *MED17* |
| Alpha-1,3-mannosyl-glycoprotein 4-beta-N-acetylglucosaminyltransferase B | Q9UQ53 | *MGAT4B* |
| HCG2019382 | Q8NEA0 | *MGC44328* |
| Mitochondrial dynamics protein MID51 | Q9NQG6 | *MIEF1* |
| Max-like protein X | Q9UH92 | *MLX* |
| Malonyl-CoA decarboxylase, mitochondrial | O95822 | *MLYCD* |
| Metallophosphoesterase 1 | Q53F39 | *MPPE1* |
| 3-mercaptopyruvate sulfurtransferase | P25325 | *MPST* |
| MpV17 transgene, murine homolog, glomerulosclerosis, isoform CRA_f | B5MC53 | *MPV17* |
| Maestro heat-like repeat-containing protein family member 2B | Q7Z745 | *MROH2B* |
| Isoform 2 of Protein MROH8 | Q9H579 | *MROH8* |
| Methionine synthase | Q99707 | *MTR* |
| Unconventional myosin-XVIIIa | MYO18A | *MYO18A* |
| Myomesin-1 | P52179 | *MYOM1* |
| N-acetylated-alpha-linked acidic dipeptidase 2 | J3KNJ3 | *NAALAD2* |
| Sialic acid synthase | Q9NR45 | *NANS* |
| Nucleosome assembly protein 1-like 3 | Q99457 | *NAP1L3* |
| NAD(P)H-hydrate epimerase | Q8NCW5 | *NAXE* |
| Nuclear receptor coactivator 5 | Q9HCD5 | *NCOA5* |
| Natural cytotoxicity triggering receptor 1 | O76036 | *NCR1* |
| NADH dehydrogenase (Ubiquinone) 1 alpha subcomplex, 13 | Q9P0J0 | *NDUFA13* |
| NADH dehydrogenase [ubiquinone] iron-sulfur protein 7, mitochondrial | O75251 | *NDUFS7* |
| Endonuclease 8-like 3 | Q8TAT5 | *NEIL3* |
| Serine/threonine-protein kinase Nek10 | Q6ZWH5 | *NEK10* |
| Sialidase-1 5 | Q99519 | *NEU1* |
| Isoform 3 of Nuclear factor of activated T-cells, cytoplasmic 2 | Q13469 | *NFATC2* |
| Niban-like protein 1 | Q96TA1 | *NIBAN2* |
| Serine/threonine-protein kinase NIM1 | Q8IY84 | *NIMIK* |
| Nipped-B-like protein | Q6KC79 | *NIPBL* |
| NF-kappa-B-repressing factor | O15226 | *NKRF* |
| NLR family CARD domain-containing protein 4 | Q9NPP4 | *NLRC4* |
| Nucleolar and coiled-body phosphoprotein 1 | Q14978 | *NOLC1* |
| NADPH oxidase 1 | Q9Y5S8 | *NOX1* |
| Nephrocysn-3 | Q7Z494 | *NPHP3* |
| Atrial natriuretic peptide receptor 2 | P20594 | *NPR2* |
| Histone-lysine N-methyltransferase NSD2 | O96028 | *NSD2* |
| Histone-lysine N-methyltransferase NSD3 | Q9BZ95 | *NSD3* |
| 5'(3')-deoxyribonucleotidase, cytosolic type | Q8TCD5 | *NT5C* |
| 5'-nucleotidase domain-containing protein 1 | Q5TFE4 | *NT5DC1* |
| Nuclear migration protein nudC | Q9Y266 | *NUDC* |
| Nuclear GTPase SLIP-GC | Q68CJ6 | *NUGGC* |
| Pyridoxal kinase | O00764 | *O00764* |
| Ornithine aminotransferase, mitochondrial | P04181 | *OAT* |
| Coiled-coil domain-containing protein 151 | A5D8V7 | *ODAD3* |
| Oral-facial-digital syndrome 1 protein | O75665 | *OFD1* |
| Optineurin | Q96CV9 | *OPTN* |
| Oxysterol-binding protein-related protein 7 | Q9BZF2 | *OSBPL7* |
| Fructose-bisphosphate aldolase C | P09972 | *ALDOC* |
| Protein disulfide-isomerase | P07237 | *P4HB* |
| Serine/threonine-protein kinase PAK 2 | Q13177 | *PAK2* |
| Paralemmin-3 | A6NDB9 | *PALM3* |
| MAGUK p55 subfamily member 5 | Q8N3R9 | *PALS1* |
| Poly [ADP-ribose] polymerase 8 | Q8N3A8 | *PARP8* |
| PAX-interacting protein 1 | Q6ZW49 | *PAXIP1* |
| Pre-mRNA cleavage complex 2 protein Pcf11 | O94913 | *PCF11* |
| Programmed cell death protein 5 | O14737 | *PDCD5* |
| Platelet-derived growth factor receptor alpha | P16234 | *PDGFRA* |
| Protein disulfide-isomerase A3 | P30101 | *PDIA3* |
| Protein disulfide-isomerase A6 | Q15084 | *PDIA6* |
| PDZ domain-containing protein 2 | O15018 | *PDZD2* |
| PDZ domain-containing protein 4 | Q76G19 | *PDZD4* |
| Pescadillo homolog | O00541 | *PES1* |
| Prohibin-2 | Q99623 | *PHB2* |
| Phosphadylinositol 4,5-bisphosphate 3-kinase catalyc subunit alpha isoform | P42336 | *PIK3CA* |
| Pyruvate kinase | Q504U3 | *PKM2* |
| Pyruvate kinase PKM | P14618 | *PKM2* |
| Serine/threonine-protein kinase N2 | Q16513 | *PKN2* |
| Plakophilin-2 | Q99959 | *PKP2* |
| Plakophilin-3 | Q9Y446 | *PKP3* |
| 1-phosphadylinositol 4,5-bisphosphate phosphodiesterase delta-1 | P51178 | *PLCD1* |
| Pleckstrin homology domain-containing family A member 7 | Q6IQ23 | *PLEKHA7* |
| Isoform 4 of Perilipin-3 | O60664 | *PLIN3* |
| Probable lipid phosphate phosphatase PPAPDC3 | Q8NBV4 | *PLPP7* |
| Paraneoplastic antigen Ma3 | Q9UL41 | *PNMA3* |
| Purine nucleoside phosphorylase | P00491 | *PNP* |
| Polyribonucleotide nucleotidyltransferase 1, mitochondrial | Q8TCS8 | *PNPT1* |
| DNA polymerase subunit gamma-1 | P54098 | *POLG* |
| DNA polymerase nu | Q7Z5Q5 | *POLN* |
| DNA-directed RNA polymerase, mitochondrial | O00411 | *POLRMT* |
| Serum paraoxonase/lactonase 3 | Q15166 | *PON3* |
| Putative beta-actin-like protein 3 | Q9BYX7 | *POTEKP* |
| Inorganic pyrophosphatase | Q15181 | *PPA1* |
| Peptidyl-prolyl cis-trans isomerase A | P62937 | *PPIA* |
| Isoform 5 of Protein phosphatase 1 regulatory subunit 12A | O14974 | *PPP1R12A* |
| Serine/threonine-protein phosphatase 4 regulatory subunit 4 | Q6NUP7 | *PPP4R4* |
| Peptidylprolyl isomerase domain and WD repeat-containing protein 1 | Q96BP3 | *PPWD1* |
| Protein regulator of cytokinesis 1 | O43663 | *PRC1* |
| PR domain-containing protein 11 (Fragment) | H3BSZ2 | *PRDM11* |
| PR domain zinc finger protein 2 | Q13029 | *PRDM2* |
| Peroxiredoxin-1 (Fragment) | A0A0A0MSI0 | *PRDX1* |
| Peroxiredoxin-6 | P30041 | *PRDX6* |
| cAMP-dependent protein kinase type II-alpha regulatory subunit | P13861 | *PRKAR2A* |
| Proline-rich basic protein 1 | E7EW31 | *PROB1* |
| Prospero homeobox protein 1 | Q92786 | *PROX1* |
| Protein PRRC1 | Q96M27 | *PRRC1* |
| Protein PRRC2A | P48634 | *PRRC2A* |
| Proline-rich transmembrane protein 3 | Q5FWE3 | *PRRT3* |
| Proteasome subunit alpha type-5 | P28066 | *PSMA5* |
| 26S protease regulatory subunit 6A | E9PM69 | *PSMC3* |
| Isoform 3 of Prostaglandin E synthase 3 | Q15185 | *PTGES3* |
| Focal adhesion kinase 1 | Q05397 | *PTK2* |
| Glutamine--tRNA ligase | P47897 | *QARS1* |
| Rab11 family-interacting protein 1 | Q6WKZ4 | *RAB11FIP1* |
| Ras-related protein Rab-30 | Q15771 | *RAB30* |
| Rab-3A-interacting protein | Q96QF0 | *RAB3IP* |
| Rab GTPase-activating protein 1-like | Q5R372 | *RABGAP1L* |
| Guanine nucleotide-binding protein subunit beta-2-like 1 | P63244 | *RACK1* |
| DNA repair protein RAD50 | Q92878 | *RAD50* |
| RAD51-associated protein 2 | Q09MP3 | *RAD51AP2* |
| Retinoic acid-induced protein 1 | Q7Z5J4 | *RAI1* |
| Rap guanine nucleotide exchange factor 1 | Q13905 | *RAPGEF1* |
| Ras-specific guanine nucleotide-releasing factor 2 | O14827 | *RASGRF2* |
| RB1-inducible coiled-coil protein 1 | Q8TDY2 | *RB1CC1* |
| Putative RNA-binding protein 15 | Q96T37 | *RBM15* |
| Splicing factor 45 | Q96I25 | *RBM17* |
| RNA-binding protein 25 | P49756 | *RBM25* |
| RNA-binding mof protein, X chromosome | P38159 | *RBMX* |
| Reculocalbin-1 | Q15293 | *RCN1* |
| Uncharacterized protein KIAA1551 | Q9HCM1 | *RESF1* |
| RE1-silencing transcription factor | Q13127 | *REST* |
| Replication factor C subunit 5 | P40937 | *RFC5* |
| Regucalcin | Q15493 | *RGN* |
| Regulator of G-protein signaling 19 | P49795 | *RGS19* |
| Regulator of G-protein signaling 3 | P49796 | *RGS3* |
| Regulator of G-protein signaling 7 | P49802 | *RGS7* |
| Mitochondrial Rho GTPase 2 | Q8IXI1 | *RHOT2* |
| RIMS-binding protein 3A | Q9UFD9 | *RIMBP3* |
| Zinc finger protein Rlf | Q13129 | *RLF* |
| Regulator of microtubule dynamics protein 2 | Q96LZ7 | *RMDN2* |
| Ribonuclease H2 subunit A | O75792 | *RNASEH2A* |
| RING finger protein 207 | Q6ZRF8 | *RNF207* |
| Rho-associated protein kinase 2 | O75116 | *ROCK2* |
| Ribosomal protein S6 kinase alpha-1 | Q15418 | *RPS6KA1* |
| Ribosomal RNA processing protein 1 homolog B | Q14684 | *RRP1B* |
| 40S ribosomal protein S15a | P62244 | *RS15A* |
| Lysine-specific demethylase 9 | Q5VWQ0 | *RSBN1* |
| Rotan | Q86VV8 | *RTTN* |
| RUN and FYVE domain-containing protein 2 | Q8WXA3 | *RUFY2* |
| Sacsin | Q9NZJ4 | *SACS* |
| Suprabasin | Q6UWP8 | *SBSN* |
| Protein SCAF11 | Q99590 | *SCAF11* |
| Sec1 family domain-containing protein 1 | Q8WVM8 | *SCFD1* |
| Sodium channel protein type 11 subunit alpha | Q9UI33 | *SCN11A* |
| Sodium channel protein type 1 subunit alpha | P35498 | *SCN1A* |
| Isoform 2 of Protein SEC13 homolog | P55735 | *SEC13* |
| Semaphorin-4D | Q92854 | *SEMA4D* |
| Semaphorin-4F | O95754 | *SEMA4F* |
| Semenogelin-2 | Q02383 | *SEMG2* |
| Sentrin-specific protease 1 | Q9P0U3 | *SENP1* |
| Sentrin-specific protease 5 | Q96HI0 | *SENP5* |
| Isoform 3 of Plasminogen activator inhibitor 1 RNA-binding protein | Q8NC51 | *SERBP1* |
| Serine incorporator 1 | Q9NRX5 | *SERINC1* |
| Leukocyte elastase inhibitor | P30740 | *SERPINB1* |
| Serpin H1 | P50454 | *SERPINH1* |
| Splicing factor 3B subunit 4 | Q15427 | *SF3B4* |
| Protein SFI1 homolog | A8K8P3 | *SFI1* |
| Splicing factor, suppressor of white-apricot homolog | Q12872 | *SFSWAP* |
| Endophilin-B1 | Q9Y371 | *SH3GLB1* |
| Isoform 2 of SH3 and PX domain-containing protein 2A] | Q5TCZ1 | *SH3PXD2A* |
| SH3 and multiple ankyrin repeat domains protein 3 | Q9BYB0 | *SHANK3* |
| Serine hydroxymethyltransferase (Fragment) | Q5BJF5 | *SHMT2* |
| Signal-induced proliferation-associated 1-like protein 1 | O43166 | *SIPA1L1* |
| Solute carrier family 15 member 3 | Q8IY34 | *SLC15A3* |
| Solute carrier family 2, facilitated glucose transporter member 14 | Q8TDB8 | *SLC2A14* |
| Sodium-dependent phosphate transport protein 2C | Q8N130 | *SLC34A3* |
| Kanadaptin | A0A087X0M4 | *SLC4A1AP* |
| Sodium/calcium exchanger 1 | P32418 | *SLC8A1* |
| Structural maintenance of chromosomes protein 6 | Q96SB8 | *SMC6* |
| Sortilin-related receptor | Q92673 | *SORL1* |
| Mitochondria-eating protein | Q8TC71 | *SPATA18* |
| Striated muscle preferentially expressed protein kinase | Q15772 | *SPEG* |
| Msx2-interacng protein | Q96T58 | *SPEN* |
| Spatacsin | Q96JI7 | *SPG11* |
| A-kinase anchor protein SPHKAP | Q2M3C7 | *SPHKAP* |
| SPRY domain-containing protein 4 | Q8WW59 | *SPRYD4* |
| Helicase SRCAP | Q6ZRS2 | *SRCAP* |
| Serine/arginine repetive matrix protein 1 | Q8IYB3 | *SRRM1* |
| Serine/arginine-rich splicing factor 1 | Q07955 | *SRRM2* |
| Serine/arginine-rich splicing factor 4 | Q08170 | *SRRM3* |
| Serine/arginine-rich-splicing factor 3 | P84103 | *SRRM4* |
| Serine/threonine-protein kinase D3 (Fragment) | O94806 | *SRRM5* |
| ST13 protein (Fragment) | Q0IJ56 | *ST13* |
| Cohesin subunit SA-1 | Q8WVM7 | *STAG1* |
| Stress-induced-phosphoprotein 1 | P31948 | *STIP1* |
| Strian-4 | Q9NRL3 | *STRN4* |
| Sulfotransferase family cytosolic 1B member 1 | O43704 | *SULT1B1* |
| Rho GTPase-activating protein SYDE2 | Q5VT97 | *SYDE2* |
| Isoform 4 of Heterogeneous nuclear ribonucleoprotein Q | O60506 | *SYNCRIP* |
| Transforming acidic coiled-coil-containing protein 3 | Q9Y6A5 | *TACC3* |
| Transgelin-2 (Fragment) | X6RJP6 | *TAGLN2* |
| Probable threonine--tRNA ligase 2, cytoplasmic | A2RTX5 | *TARS3* |
| TBC1 domain family member 10B | Q4KMP7 | *TBC1D10B* |
| Transducin beta-like protein 2 | E9PF19 | *TBL2* |
| Transcription elongation factor A protein-like 1 | Q15170 | *TCEAL1* |
| Trichohyalin-like protein 1 | Q5QJ38 | *TCHHL1* |
| Treacle protein | Q13428 | *TCOF1* |
| T-complex protein 1 subunit alpha | P17987 | *TCP1* |
| Tudor domain-containing protein 3 | Q9H7E2 | *TDRD3* |
| Thyrotrophic embryonic factor (Fragment) | E0ZS57 | *TEF* |
| Methylcytosine dioxygenase TET3 | O43151 | *TET3* |
| Inactive serine/threonine-protein kinase TEX14 | Q8IWB6 | *TEX14* |
| Tess-expressed sequence 15 protein | Q9BXT5 | *TEX15* |
| Transforming growth factor beta receptor type 3 | Q03167 | *TGFBR3* |
| Protein meless homolog | Q9UNS1 | *TIMELESS* |
| Tight junction protein Z | Q9UDY2 | *TJP2* |
| Talin-1 | Q9Y490 | *TLN1* |
| Talin-2 | Q9Y4G6 | *TLN2* |
| Transmembrane protein 232 | C9JQI7 | *TMEM232* |
| 182 kDa tankyrase-1-binding protein | Q9C0C2 | *TNKS1BP1* |
| Trinucleode repeat-containing gene 18 protein | O15417 | *TNRC18* |
| Tumor protein 63 | Q9H3D4 | *TP63* |
| Triosephosphate isomerase isoform 1 | P60174 | *TPI1* |
| Transcription intermediary factor 1-alpha | O15164 | *TRIM24* |
| Isoform 2 of Transcription intermediary factor 1-beta | Q13263 | *TRIM28* |
| Short transient receptor potential channel | P48995 | *TRPC1* |
| Short transient receptor potential channel 6 | Q9Y210 | *TRPC6* |
| TSC22 domain family protein 2 | O75157 | *TSC22D2* |
| Teashirt homolog 3 | Q63HK5 | *TSHZ3* |
| Tess-specific serine kinase substrate | Q9UJT2 | *TSKS* |
| Thiosulfate sulfurtransferase | Q16762 | *TST* |
| Tetratricopeptide repeat protein 14 | Q96N46 | *TTC14* |
| Transcription termination factor 2 | Q9UNY4 | *TTF2* |
| Tubulin polyglutamylase TTLL6 | Q8N841 | *TTLL6* |
| Probable tubulin polyglutamylase TTLL9 | Q3SXZ7 | *TTLL9* |
| Tubulin alpha-1B chain | P68363 | *TUBA1B* |
| Tubulin, beta 2C, isoform CRA_b | Q8N6N5 | *TUBB2C* |
| Tubulin beta-4B chain | P68371 | *TUBB4B* |
| Elongation factor Tu, mitochondrial | P49411 | *TUFM* |
| Terminal uridylyltransferase 4 | Q5TAX3 | *TUT4* |
| Thioredoxin domain-containing protein 5 | Q8NBS9 | *TXNDC5* |
| Thioredoxin-like protein 1 (Fragment) | K7EML9 | *TXNL1* |
| Uveal autoantigen with coiled-coil domains and ankyrin repeats | Q9BZF9 | *UACA* |
| Ubiquitin-associated protein 1-like | F5GYI3 | *UBAP1L* |
| Polyubiquitin-C | P0CG48 | *UBC* |
| UDP-glucose 6-dehydrogenase | O60701 | *UGDH* |
| UDP-glucose:glycoprotein glucosyltransferase 1 | Q9NYU2 | *UGGT1* |
| Uridine phosphorylase 1 | Q16831 | *UPP1* |
| Ubiquinol-cytochrome-c reductase complex assembly factor 2 | Q9BRT2 | *UQCC2* |
| Ubiquitin carboxyl-terminal hydrolase 34 | Q70CQ2 | *USP34* |
| Ubiquitin carboxyl-terminal hydrolase 35 | Q9P2H5 | *USP35* |
| Isoform 1 of Vinculin | P18206 | *VCL* |
| Protein-lysine methyltransferase METTL21D | Q9H867 | *VCPKMT* |
| Voltage-dependent anion-selective channel protein 1 | P21796 | *VDAC1* |
| Villin-1 | P09327 | *VIL1* |
| Vacuolar protein sorting-associated protein 11 homolog | Q9H270 | *VPS11* |
| Isoform 3 of Syndetin | Q96JG6 | *VPS50* |
| Von Willebrand factor A domain-containing protein 3A | A6NCI4 | *VWA3A* |
| Von Willebrand factor D and EGF domain-containing protein | Q8N2E2 | *VWDE* |
| WD repeat-containing protein 3 | Q9UNX4 | *WDR3* |
| WD repeat-containing protein 87 | Q6ZQQ6 | *WDR87* |
| Wilms tumor protein 1-interacting protein | A6NIX2 | *WTIP* |
| Protein WWC2 | Q6AWC2 | *WWC2* |
| NEDD4-like E3 ubiquitin-protein ligase WWP2 | O00308 | *WWP2* |
| Xin actin-binding repeat-containing protein 2 | A4UGR9 | *XIRP2* |
| Probable ATP-dependent RNA helicase YTHDC2 | Q9H6S0 | *YTHDC2* |
| 14-3-3 protein epsilon | P62258 | *YWHAE* |
| Zinc finger and BTB domain-containing protein 12 | Q9Y330 | *ZBTB12* |
| Zinc finger and BTB domain-containing protein 21 | Q9ULJ3 | *ZBTB21* |
| DBF4-type zinc finger-containing protein 2 | Q9HCK1 | *ZDBF2* |
| AN1-type zinc finger protein 4 | Q86XD8 | *ZFAND4* |
| Zinc finger protein 92 homolog | A6NM28 | *ZFP92* |
| Zinc finger protein 197 | O14709 | *ZNF197* |
| Zinc finger protein 254 | O75437 | *ZNF254* |
| Zinc finger protein 263 | O14978 | *ZNF263* |
| Zinc finger protein 318 | Q5VUA4 | *ZNF318* |
| Zinc finger protein 407 | Q9C0G0 | *ZNF407* |
| Zinc finger protein 658 | Q5TYW1 | *ZNF658* |
| Zinc finger protein 658B | Q4V348 | *ZNF658B* |
| Zinc finger protein 692 | Q9BU19 | *ZNF692* |
| Zinc finger protein 732 | B4DXR9 | *ZNF732* |
| Zinc finger protein 800 | Q2TB10 | *ZNF800* |
| Zinc finger protein 831 | Q5JPB2 | *ZNF831* |
| Zinc finger Ran-binding domain-containing protein 2 | O95218 | *ZRANB2* |
| Zinc finger and SCAN domain-containing protein 1 | Q8NBB4 | *ZSCAN1* |
| Putative zinc finger and SCAN domain-containing protein 5D | P0CG00 | *ZSCAN5DP* |
| Protein zyg-11 homolog A | Q6WRX3 | *ZYG11A* |
| Zinc finger ZZ-type and EF-hand domain-containing protein 1 | O43149 | *ZZEF1* |
| Protein ID not associated with a gene | | |
| B4DJX1, B4DZ08, Q9BRL5, B3KML9, Q8TCH5, Q6ZPA5, B3KPS3, Q8N8C3, B4DH02, B4DM33, B4DP56, B4DNE0, B7Z2X4, B7Z4Z6, B4DMA2, B4DVY2, B4DI39, B4E388, B4DRH6, B4DUQ1, B7Z4F6, B4E022, B4DVQ0, B4DQ52, A8K5X8, B3KQF5, B2R6U8, B2R9I9, A8K9A9, B4DW52, B4DMF5, Q53HF2, Q7L4M3, O00370, Q7Z757, Q71V99, Q53FV0, B3KQT9, Q59H19, A8MWP4, E7D7X9, B7Z2F4 and B7Z9L0. | | |

**Supplementary Table 2.** List of proteins used for bioinformatic analysis.

| **Description** | **UniProt ID** | **Gen** | **Reference** |
| --- | --- | --- | --- |
| Actin, aortic smooth muscle | P62736 | *ACTA2* | ^19^ |
| Actin, cytoplasmic 1 | P60709 | *ACTB* | ^17-19^ |
| Adenosylhomocysteinase | P23526 | *AHCY* | ^18,19^ |
| Ankyrin repeat domain-containing protein 33B | A6NCL7 | *ANKRD33B* | ^18,19^ |
| Annexin A2 | P07355 | *ANXA2* | ^18,19^ |
| Annexin A4 | P09525 | *ANXA4* | ^16-18^ |
| ATP synthase subunit alpha, mitochondrial | P25705 | *ATP5F1A* | ^18,19^ |
| Centromere protein J | Q9HC77 | *CENPJ* | ^19^ |
| Liver carboxylesterase 1 | P23141 | *CES1* | ^16-18^ |
| Cilia- and flagella-associated protein 54 | Q96N23 | *CFAP54* | ^19^ |
| Rootletin | Q5TZA2 | *CROCC* | ^19^ |
| Dachshund homolog 2 | Q96NX9 | *DACH2* | ^19^ |
| DEP domain-containing protein 1A | Q5TB30 | *DEPDC1* | ^19^ |
| Elongation factor 2 | P13639 | *EEF2* | ^16,18,19^ |
| Alpha-enolase | P06733 | *ENO1* | ^18,19^ |
| Epidermal growth factor receptor substrate 15-like 1 | Q9UBC2 | *EPS15L1* | ^18,19^ |
| ERC protein 2 | O15083 | *ERC2* | ^18,19^ |
| FLJ44955 protein | Q0VFX3 | *FLI44955* | ^19^ |
| Filamin-B | O75369 | *FLNB* | ^18,19^ |
| FOXL2 neighbor protein | Q6ZUU3 | *FOXL2NB* | ^19^ |
| Polypeptide N-acetylgalactosaminyltransferase 9 | Q9HCQ5 | *GALNT9* | ^19^ |
| Glyceraldehyde-3-phosphate dehydrogenase | P04406 | *GAPDH* | ^18,19^ |
| Glutamate dehydrogenase 1, mitochondrial | P00367 | *GLUD1* | ^16,18^ |
| Aspartate aminotransferase | A0A024R6W0 | *GOT2* | ^18,19^ |
| Glutathione synthetase | P48637 | *GSS* | ^17-19^ |
| Hemicentin-1 | Q96RW7 | *HMCN1* | ^19^ |
| Heat shock 70 kDa protein 1A | P0DMV8 | *HSPA1A* | ^16,18,19^ |
| Insulin-like growth factor 2 mRNA-binding protein 1 | Q9NZI8 | *IGF2BP1* | ^18,19^ |
| Keratin, type I cytoskeletal 18 | P05783 | *KRT18* | ^16,19^ |
| Keratin, type II cytoskeletal 8 | P05787 | *KRT8* | ^16,19^ |
| Low-density lipoprotein receptor-related protein 1B | Q9NZR2 | *LRP1B* | ^19^ |
| Mediator of RNA polymerase II transcription subunit 17 | Q9NVC6 | *MED17* | ^19^ |
| NADH dehydrogenase (Ubiquinone) 1 alpha subcomplex, 13 | Q9P0J0 | *NDUFA13* | ^18,19^ |
| Histone-lysine N-methyltransferase NSD3 | Q9BZ95 | *NSD3* | ^19^ |
| Fructose-bisphosphate aldolase C | P09972 | *ALDOC* | ^17,18^ |
| Protein disulfide-isomerase A3 | P30101 | *PDIA3* | ^16,17,19^ |
| Inorganic pyrophosphatase | Q15181 | *PPA1* | ^18,19^ |
| Peroxiredoxin-6 | P30041 | *PRDX6* | ^17,19^ |
| Proteasome subunit alpha type-5 | P28066 | *PSMA5* | ^18,19^ |
| Sentrin-specific protease 1 | Q9P0U3 | *SENP1* | ^19^ |
| Serpin H1 | P50454 | *SERPINH1* | ^18,19^ |
| Protein SFI1 homolog | A8K8P3 | *SFI1* | ^19^ |
| Solute carrier family 2, facilitated glucose transporter member 14 | Q8TDB8 | *SLC2A14* | ^18,19^ |
| Msx2-interacng protein | Q96T58 | *SPEN* | ^18,19^ |
| Transforming growth factor beta receptor type 3 | Q03167 | *TGFBR3* | ^18,19^ |
| Elongation factor Tu, mitochondrial | P49411 | *TUFM* | ^18,19^ |
| Zinc finger protein 658B | Q4V348 | *ZNF658B* | ^19^ |
