# Supplementary figures and images for: Bioinformatic Analysis of the Effect of Silver Nanoparticles on Colorectal Cancer Cell Line
Source: Biomed Res Int. 2022 Apr 11;2022:6828837. doi: 10.1155/2022/6828837 (PMC9015850; doi:10.1155/2022/6828837)

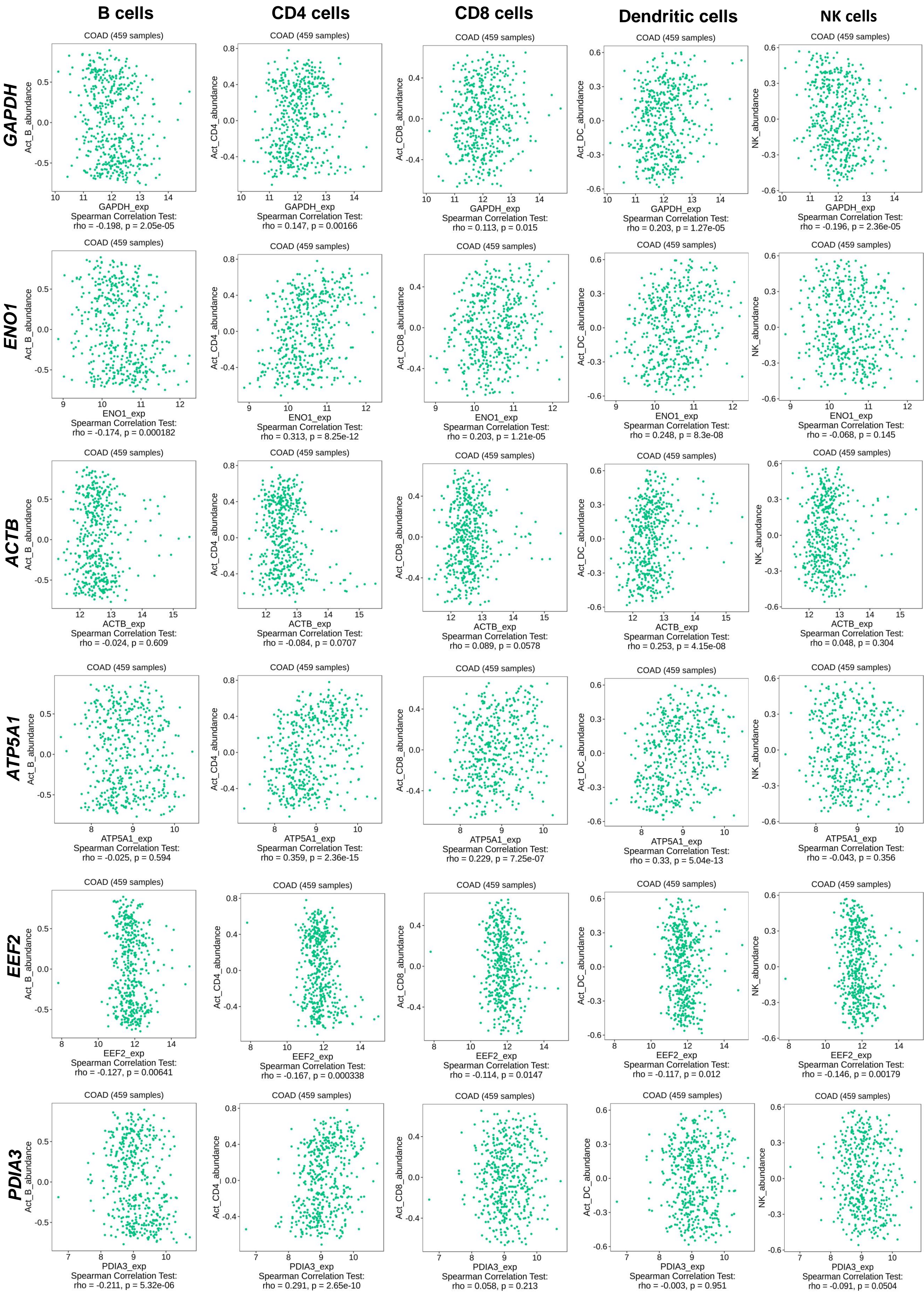

Supplement: Supplementary Materials — The supplementary material contains the list of proteins obtained from the research work used in this work. Likewise, correlation plots between hub genes and immune cells for COAD and READ are shown in supplementary Figures 1 and 2, respectively. [file 6828837.f1.zip › Supplementary Figure 1.pdf]

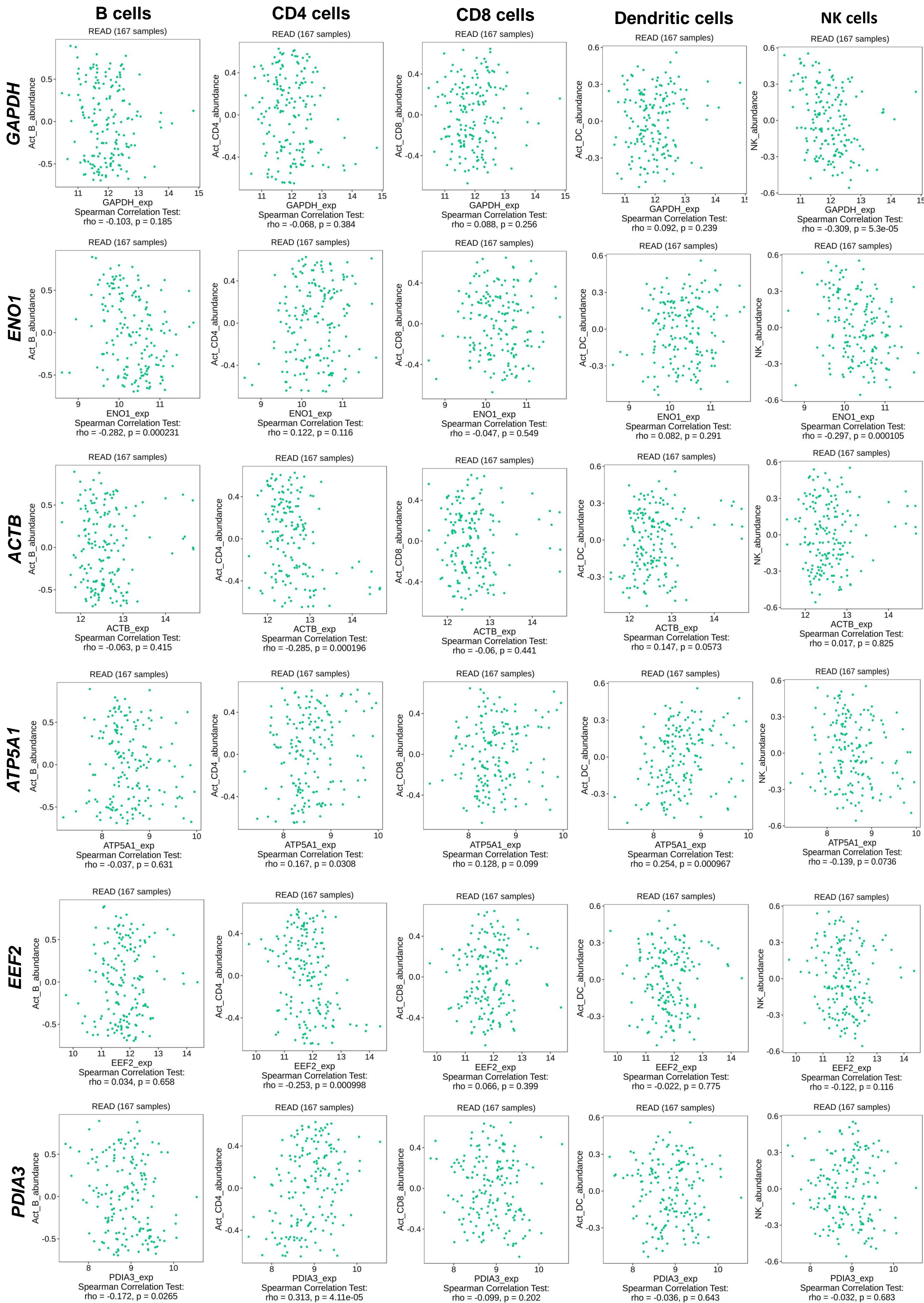

Supplement: Supplementary Materials — The supplementary material contains the list of proteins obtained from the research work used in this work. Likewise, correlation plots between hub genes and immune cells for COAD and READ are shown in supplementary Figures 1 and 2, respectively. [file 6828837.f1.zip › Supplementary Figure 2.pdf]
